# FGFR2c Upregulation Contributes to Cancer-Associated Fibroblast Program Activation and to Enhanced Autophagy in Actinic Keratosis-Derived Dermal Fibroblasts: A Possible Role in Precancerous Cell/Stromal Cell Crosstalk

**DOI:** 10.3390/biology12030463

**Published:** 2023-03-16

**Authors:** Luisa Guttieri, Salvatore Raffa, Gerardo Salerno, Rachele Bigi, Flavia Persechino, Vincenzo Visco, Maria Rosaria Torrisi, Danilo Ranieri, Francesca Belleudi

**Affiliations:** 1 Department of Clinical and Molecular Medicine, Sapienza University of Rome, 00161 Rome, Italy; luisa.guttieri@uniroma1.it (L.G.); salvatore.raffa@uniroma1.it (S.R.); flaviapersechino@hotmail.it (F.P.); vincenzo.visco1@uniroma1.it (V.V.); mara.torrisi@uniroma1.it (M.R.T.); danilo.ranieri@uniroma1.it (D.R.); 2 Laboratory of Ultrastructural Pathology, Unit of Medical Genetics and Advanced Cellular Diagnostics, Department of Diagnostic Sciences, Sant’Andrea University Hospital, 00161 Rome, Italy; 3 Department of Neurosciences, Mental Health and Sensory Organs (NESMOS), Sapienza University of Rome, 00161 Rome, Italy; gerardo.salerno@uniroma1.it (G.S.); rachele.bigi@uniroma1.it (R.B.); 4 Laboratory of Clinical Chemistry, Sant’Andrea University Hospital, 00161 Rome, Italy

**Keywords:** FGFR2, autophagy, actinic keratosis, CAF

## Abstract

**Simple Summary:**

In this work, we checked the possible tumor-promoting role of the mesenchymal FGFR2c isoform when overexpressed in AK stromal fibroblasts. We found that already starting from the early AK lesions, the dermal expression of FGFR2c is upregulated and accompanied by: (i) the repression of the CAF transcription repressor CSL, (ii) the upregulation of the CAF activator ULK3, and (iii) the induction of CAF genes. Immunofluorescence and molecular analysis coupled with silencing approaches suggested that, in primary cultures of KIN I-derived fibroblasts, the upregulation of FGFR2c contribute to the CAF signature and the increased autophagy in response to FGF2. Magnetic bead-based multiplex assay combined with FGFR tyrosine kinase inhibitors suggested that the enhanced secretion of IL-6, especially in response to FGF2, could be attributable to FGFR2c increased expression and signaling, possibly via the establishment of FGFR2c-mediated secretory autophagy. Overall, our results suggest that FGFR2c could be a signaling molecule contributing to precancerous/stromal cell oncogenic crosstalk, indicating it as a possible early molecular marker predictive for AK malignant progression.

**Abstract:**

Actinic keratosis (AK) is a preneoplastic skin disorder which can rapidly progress to cutaneous squamous cell carcinomas (SCCs). In light of our previous findings, indicating a possible oncogenic role of the mesenchymal isoform of FGFR2 (FGFR2c) aberrantly expressed in AK keratinocytes, we analyzed the possible tumor-promoting role of this receptor in the stromal AK counterpart in this work. Molecular analysis showed that, particularly in early AK lesions, FGFR2c dermal upregulation is accompanied by the downregulation of the cancer-associated fibroblasts (CAF) transcription repressor CSL, the upregulation of the CAF activator ULK3, and the consequent CAF gene induction. Immunofluorescence and molecular analysis, coupled with silencing approaches by siRNA, applied on primary cultures of KIN I-derived fibroblasts, indicated that FGFR2c upregulation contribute to CAF signature and the increased autophagy in response to FGF2. Magnetic bead-based multiplex assay, combined with FGFR2 signaling shut-off approaches, indicated that, especially in response to FGF2, IL-6 secretion could depend on FGFR2c high expression and signaling, suggesting the possible establishment of FGFR2c-dependent secretory autophagy, contributing to tumor-promoting factor release. Overall, our results identified FGFR2c as a signaling molecule involved in controlling precancerous/stromal cell oncogenic crosstalk, pointing to this receptor as a possible early molecular marker predictive for AK’s rapid malignant progression.

## 1. Introduction

Actinic keratosis (AK) is a skin disorder associated with UV exposure, presenting characteristic morphological and functional alterations of the keratinocytes and displaying a rare progression to squamous cell carcinoma (SCC) [1,2]. AK progression is described by three grades: keratinocytic intraepithelial neoplasia of low grade (KIN I), ‘intermediate grade (KIN II), and high grade (KIN III) [3,4]. Less frequently, some KIN I lesions can directly progress toward aggressive SCCs via the “differentiated” pathway [3,5], characterized by hallmarks, among which one of the most typical is the epithelial-mesenchymal transition (EMT) [6].

Accumulating findings pointed to the dysregulation of the fibroblast growth factor receptor (FGFR) axis as an additional event involved in the dysregulation of keratinocyte functions accompanying AK onset and possibly progression [7,8,9]. In line with this possibility, we previously demonstrated an increased degradation of the epithelial variant of FGFR2 (FGFR2b) in epidermal keratinocytes exposed to UV [10], which is accompanied by increased apoptosis in these cells [11]. Our further studies have indicated that FGFR2b downregulation could be part of a more articulated molecular event (the FGFR2 isoform switch), which leads to the appearance of the mesenchymal variant FGFR2c. We have previously demonstrated a key role for this receptor in inducing alteration of keratinocyte differentiation and stratification [12], as well as EMT and invasive behavior [13,14,15], which are all hallmarks of tumor malignant progression. In line with this evidence, our more recent work, aimed to identify specific keratinocyte gene expression profiles predictive for AK malignant progression, indicated that modulation of FGFR2 epithelial and mesenchymal variants compatible with FGFR2 altered splicing could be an early event possibly involved in KIN I progression directly to SCC [15]. In addition, an enhanced expression of FGF2 has been found in the dermal portion of the analyzed AK lesions, indicating an increased ability of AK stromal cells to possibly “feed” aberrant epidermal/dermal loops based on the FGF2/FGFRc axis [15]. Indeed, an essential role for dysregulated crosstalk between tumor cells and cancer-associated fibroblasts (CAF) has been extensively proposed in several tumors [16,17], including SCC [18,19]. In the specific case of the advanced HNSCC, it has been demonstrated that the secretion of FGF2 by tumor cells induces the upregulation of the FGF2/FGFR2 axis in dermal CAFs, which, in turn, contributes to CAF activation and mTOR-mediated enhancement of autophagy, a tumor-promoting event involved in the secretion of pro-tumorigenic factors [20].

Actually, it has been proposed that, at least in some cases which could possibly coincide with those AK lesions rapidly progressing in SCC, dermal fibroblast dysregulations may not only sustain but even precede the development of skin cancers. In these cells, the inactivation of the Notch-dependent CAF transcription repressor CSL promotes a CAF-like state, which stimulates the proliferation of the overlying epidermal keratinocyte, favoring the onset of precancerous AK [16,17]. What remains to be elucidated is whether dysregulation of FGFR2c expression can play any role in these events.

To address this topic, in this work, we started with the analysis of FGFR2c expression in the dermal portion of differently graded AK lesions, paying particular attention to the KIN I trend and searching for a possible link with CAF and autophagic signature. Taking advantage of the use of the in vitro model of KIN I-derived HFs, we analyzed the possible direct contribution of FGFR2c, expressed in these stromal cells, to the activation of both the CAF-like phenotype and autophagy, two tumor-promoting events which could, in turn, contribute to a rapid AK malignant progression.

## 2. Materials and Methods

### 2.1. Institutional Review Board Statement

All subjects involved in this study gave their informed consent. The study complies with the Declaration of Helsinki and was approved by The Institutional Review Board of “Sapienza” University and Sant’Andrea Hospital (protocol n° 176/2011).

### 2.2. Histological Samples

Histological samples were obtained from differently graded AK lesions and corresponding perilesional areas (PL): KIN I; *n* = 3; KIN II *n* = 1 and KIN III *n* = 2. Skin biopsies of AK and perilesional areas were taken using punch 0.5 mm. Biopsies were taken at 0.5 cm inside (for lesional samples) and 1 cm outside (for perilesional samples) the margin of lesions clinically compatible with the diagnosis of actinic keratosis (AK). This protocol has been adopted as the distance of 1 cm is considered suitable to minimize the risk of sampling perilesional specimens within the cancerization fields. All samples were from patients of the Dermatology Unit of the Sant’Andrea Hospital of Rome. Patients were extensively informed, as previously described [15]. Samples were marked using a numeric code (e.g., #1PL, #1AK). The procedure for epidermal/dermal separation has been previously described [15]. Dermal portions were used for total RNA extraction and to obtain primary cultures of human fibroblasts.

### 2.3. Primary Cell Cultures and Treatments

Primary cultures of fibroblasts were isolated from dermal portion as described [15] and cultured in Dulbecco’s modified Eagle’s medium (DMEM, Corning, New York, NY, USA), supplemented with 10% fetal bovine serum (FBS, Cytiva, Logan, UT, USA) plus penicillin, streptomycin, and glutamine. For RNA interference and consequent FGFR2c-specific silencing, cells were transfected with FGFR2c siRNA (sequence 5′-GGAATGTAACTTTTGAGGA-3′, Qiagen, Hilden, Germany) or with a control sequence Cx siRNA (5′-AATTCTCCGAACGTGTCACGT-3′) (Qiagen, Hilden, Germany), using Lipofectamine 2000 Transfection Reagent (Invitrogen, Waltham, MA, USA) according to the manufacturer’s protocol. For growth factor stimulation and/or for inhibition of FGFR2c tyrosine kinase activity, cells were left untreated or incubated with FGF2 (PeproTech, London, UK; BFGF 100 188) and with SU5402 (Calbiochem, San Diego, CA, USA) as previously reported [14].

### 2.4. Immunofluorescence

Primary cultures of Human Fibroblasts (HFs) were grown on coverslips and processed as previously reported [14]. Cells were then incubated with the primary mouse monoclonal anti-LC3 (1:100 in PBS, 5F10 Nanotools, Teningen, Germany) for 1 h at 25 °C and then with a goat anti-mouse IgG-Alexa Fluor 488 (1:200 in PBS, Life Technologies, Carlsbad, CA, USA) for 30 min at 25 °C. Nuclei were stained with 4′,6-diamidino-2-phenylindole (DAPI) (1:1000 in PBS; Sigma-Aldrich, St Louis, MO, USA). Coverslips were finally mounted with Mowiol solution (Sigma-Aldrich, St Louis, MO, USA). Fluorescence signals were analyzed by scanning cells in a series of sequential sections with an ApoTome System (Zeiss), image analysis was performed by the Axiovision software (Zeiss), and images were obtained by 3D reconstruction of all the serial optical sections. Quantitative analysis of LC3-positive dots per cell was performed using ImageJ software [21], analyzing 100 cells for each sample in 5 different microscopy fields from 3 different experiments (*n* = 3). Results are shown as means ± standard deviation (SD). Student’s t-test was performed, and significance levels have been defined as *p* < 0.05.

### 2.5. Western Blot Analysis

Cells were lysed and protein samples were extracted as previously reported [14]. A range of 20 to 50 µg of total protein was resolved under reducing conditions by 8 or 12% SDS-PAGE and transferred to reinforced nitrocellulose (Invitrogen Waltham, MA, USA). The membranes were blocked with 5% nonfat dry milk (Bio-Rad Laboratories, Hercules, CA, USA) in PBS 0.1% Tween 20 (Bio-Rad Laboratories, Hercules, CA, USA) and incubated with anti-LC3 (Cell Signaling, Danvers, MA, USA 3868S), anti-SQSTM1 (BD Bioscience, San Josè, CA, USA, 610 833), anti-p-MTOR (Ser 2448; #5536S) monoclonal antibodies or anti-Bek (Santa Cruz Biotechnology, Santa Cruz, CA, USA; C17, sc-122), anti-p-S6K (ser 371, Cell Signaling, #9202S), anti-HSP90 (Proteintech Inc., Rosemont, IL, USA, 13171-1-AP), and polyclonal antibodies, followed by enhanced chemiluminescence (ECL) detection (Thermo Scientific, Waltham, MA, USA; 34580). The membranes were rehydrated by washing in PBS/Tween-20, stripped with 100 mM mercaptoethanol and 2% SDS for 30 min at 55 °C and probed again with anti-GAPDH (Santa Cruz Biotechnology, Santa Cruz, CA, USA; sc-32233), anti-α/β-Tubulin (Cell Signaling, #2148S), anti-S6K (Cell Signaling #9208S) polyclonal antibodies or anti-MTOR (Cell Signaling, 2983S), and anti-ACTB (Sigma-Aldrich, St Louis, MO, USA; A5441) monoclonal antibodies to estimate the protein equal loading. Densitometric analysis was performed using Quantity One Program version 4.6.8 (Bio-Rad Laboratories, Hercules, CA, USA). The resulting values from 3 independent experiments (*n* = 3) were normalized, then expressed as fold increase compared to the control value and reported in graph as mean values ± SD. Student’s t-test was performed, with significance levels defined as *p* values < 0.05.

### 2.6. Primers

Oligonucleotide primers for target genes and the housekeeping gene were chosen with the assistance of the Oligo 5.0 computer program (National Biosciences, Plymouth, MN, USA) or the online tool Primer-BLAST [22] and purchased from Invitrogen. The following primers were used: for FGFR2b/KGFR target gene: 5′-CGTGGAAAAGAACGGCAGTAAATA-3′ (sense), 5′-GAACTATTTATCCCCGAGTGCTTG-3′ (antisense); for FGFR2c target gene: 5′-TGAGGACGCTGGGGAATATACG-3′ (sense), 5′-TAGTCTGGGGAAGCTGTAATCTCCT-3′ (antisense); for CSL target gene: 5′-CAGCGCCTTCAACAGGTTTC-3′ (sense), 5′-GGACGTACGTGGAGACTGC-3′ (antisense); for ULK3 target gene: 5′-CTGGACGGCTTCATCCTCAC-3′ (sense), 5′-TCACGAGTGTCCTTCTTGGC-3′ (antisense); for TNC target gene: 5′-GGAGGGGACCACGCTGAGGT-3′ (sense), 5′-TCCCGGCCTCAGACCTGTGAG-3′ (antisense); for COX-2 target gene: 5′-CAAATTGCTGGCAGGGTTGC-3′ (sense), 5′-AGGGCTTCAGCATAAAGCGT-3′ (antisense); for ACTA2/α-SMA target gene: 5′-GCACCCCTGAACCCCAAG-3′ (sense), 5′-ACGATGCCAGTTGTGCGT-3′ (antisense); for BECN1 target gene: 5′-GGATGGTGTCTCTCGCAGAT-3′ (sense), 5′-TTGGCACTTTCTGTGGACAT-3′ (antisense); for ATG5 target gene: 5′-CAACTTGTTTCACGCTATATCAGG-3′ (sense), 5′-CACTTTGTCAGTTACCAACGTCA-3′ (antisense); for MAP1LC3B/LC3 target gene: 5′-CGCACCTTCGAACAAAGAG-3′ (sense), 5′-CTCACCCTTGTATCGTTCTATTATCA-3′ (antisense); for FGF2 target 5′-ATGGCAGCCGGGAGCATCACCCACG-3′ (sense), 5′-TCAGCTCTTCGCAGACATTGGAAG-3′ (antisense); for IL-6 target 5′-TGAAAGCAGCAAAGAGGCAC-3′ (sense), 5′-CACCAGGCAAGTCTCCTCAT-3′ (antisense); for FGFR1c target gene 5′-TGGGAGCATTAACCACACCTACC-3′ (sense), 5′- GCACCTCCATTTCCTTGTCG-3′ (antisense); for FGFR3c target gene 5′-CGCCCTACGTCACTGTACTCAA-3′ (sense), 5′-GTGACATTGTGCAAGGACAGAAC-3′ (antisense); for FGFR4 target gene 5′-CTGTGGCCGTCAAGATGCTCAA-3′ (sense), 5′-ATGTTCTTGTGTCGGCCGATCA-3′ (antisense); for the 18S rRNA housekeeping gene 5′-CGAGCCGCCTGGATACC-3′ (sense), and 5′-CATGGCCTCAGTTCCGAAAA-3′ (antisense). For each primer pair, no-template control and no-reverse-transcriptase control (RT negative) assays were performed since they produced negligible signals.

### 2.7. RNA Extraction and cDNA Synthesis

RNA was extracted using the TRIzol method (Invitrogen, Waltham, MA, USA) and processed as reported [12]. Then, RNA was used to reverse transcription using iScriptTM cDNA synthesis kit (Bio-Rad Laboratories, Hercules, CA, USA) according to manufacturer’s instructions.

### 2.8. PCR Amplification and Real-Time Quantitation

Real-time PCR was performed, as described [13]. Real-time quantitation was performed with the help of the iCycler IQ optical system software version 3.0a (Bio-Rad), according to the manufacturer’s manual. Threshold cycle values were calculated using the Pfaffl method and specificity of PCR products was verified by melting curve analysis. The relative expression of the housekeeping gene was used for standardizing the reaction. Value of the target gene FGFR2c was normalized with respect to the value of primary culture of HFs isolated from a dermal perilesional sample. Value of the target gene FGFR2b was normalized with respect to the value of the human keratinocyte cell line HaCaT. Values of all the other target genes were normalized with respect to the mean of values obtained by all dermal perilesional samples. The mRNA levels are expressed as the mean ± SD from three independent experiments (*n* = 3) in triplicate. Student’s t-test was performed, with significance levels defined as *p* values < 0.05.

### 2.9. Magnetic Bead-Based Multiplex Assay

Supernatants of cells treated as above were collected after 72 h of culture in 1.5 mL centrifuge tubes and then centrifuged at 14,000 rpm for 10 min. The supernatants were then transferred into new 1.5 mL centrifuge tubes and stored at −20 °C. Then, Interleukin IL-6 was determined using a human cytokine/chemokine magnetic bead panel A (Milliplex MAP kit, Millipore Corp., Billerica, MA, USA) and measured by a Magpix Luminex instrument and Xponent software (version 4.2, Luminex Corp, Austin, TX, USA) according to manufacturer’s instruction for supernatants. The results were expressed in pg/mL and reported as mean values ± SD from three independent experiments. Student’s t-test was performed, with significance levels defined as *p* values < 0.05.

### 2.10. Statistical Analysis

For clinical samples, the statistical analysis of relative expression rates of AK versus PL portion, the Wilcoxon rank-sum test for dependent samples was applied. All the results were expressed as the mean ± SD, and significance level was defined as *p* < 0.05.

## 3. Results

### 3.1. FGFR2c Upregulation in AK Samples, including KIN I, Is Accompanied by the CSL/ULK3 Loop Modulation and CAF Gene Increase, as Well as by the Transcriptional Induction of Key Autophagic Genes

**Figure 1:**
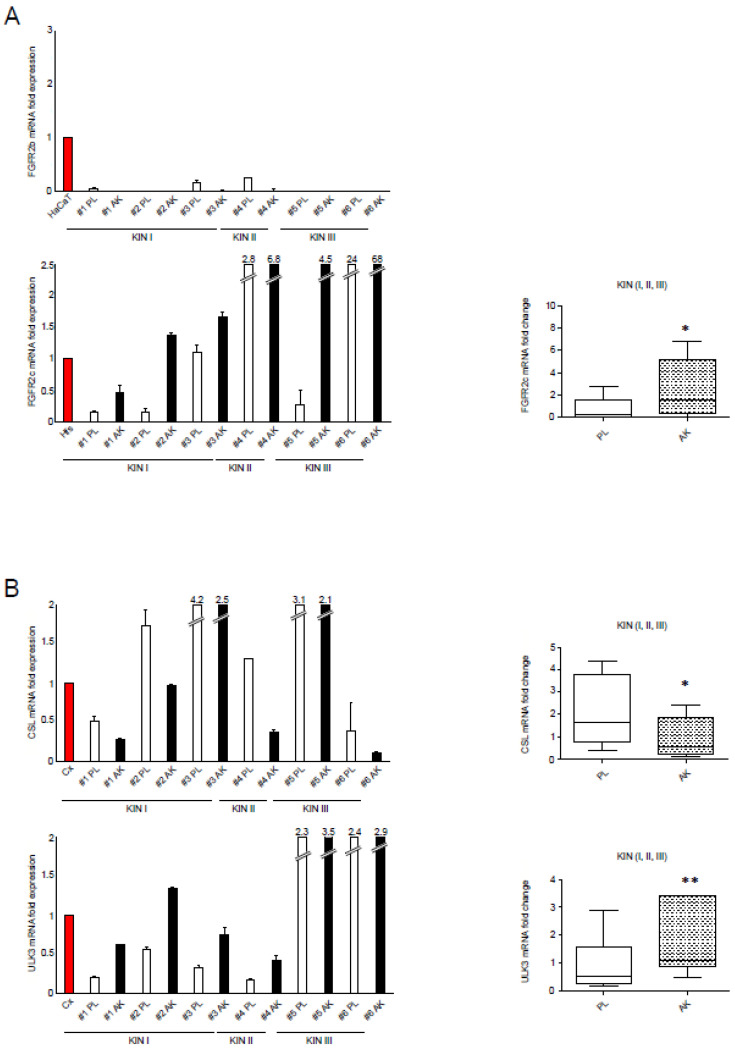
Expression of FGFR2 isoforms and CAF gene regulators CSL and ULK3 in the dermal portion of KIN I, KIN II, and KIN III samples. Epidermal FGFR2b isoform (**A**), mesenchymal FGFR2c isoform (**A**), CSL (**B**), and ULK3 (**B**) mRNA levels were evaluated by real-time RT-PCR in KIN I, KIN II, and KIN III samples (PL—perilesional samples, white bars; AK—lesional samples, black bars) and normalized respect to HaCaT cells, HFs or the mean of all the perilesional values (Cx, red bar) as reported in the graph. Results are expressed as mean ± standard deviation (SD). Statistical analysis of relative expression rates of KIN I + KIN II + KIN III AK versus KIN + KIN I + KIN III PL samples was performed using Wilcoxon rank-sum test, and significance levels were defined as *p* < 0.05. * *p* < 0.05; ** *p* < 0.01 versus the corresponding PL samples.

Based on the evidence that the upregulation of FGFR2 in dermal fibroblasts has been recognized as a hallmark for the stroma-mediated HNSCC malignant progression [20], we wondered if modulation of this receptor could be also detected in precancerous AK lesions and if this modulation could possibly contribute to AK progression. To answer this question, we first assessed the expression of the two FGFR2 tissue-specific isoforms, comparing their levels in the dermal portion of lesional (AK) KIN I, KIN II, and KIN III samples with those detected in the same portion of corresponding perilesional (PL) controls. Real-time RT-PCR analysis showed that, while all lesional and perilesional samples displayed quite an undetectable expression of the epithelial FGFR2b isoform (Figure 1A, upper panel), as expected in a stromal context, the expression of the mesenchymal FGFR2c variant appeared clearly increased in all differently graded AK lesions respect to the corresponding controls (Figure 1A, lower panel). This trend was evident already starting from KIN I samples (Figure 1A, lower panel). The statistical analysis performed by the Wilcoxon test for dependent samples comparing the mean values of the entire cohort of AK lesions versus their controls confirmed that the observed increase of FGFR2c was statistically significant (Figure 1A, right panel).

It has been reported that the CAF signature of stromal fibroblasts is driven by the downregulation of the notch-related transcription factor repressor CSL [8,16,17], which sometimes precedes the AK onset [16,17]. Since this event is accompanied by FGF2 upregulation in both epithelial and stroma cells, suggesting its possible dependence on FGF/FGFR axis dysregulation [8], we wondered whether the observed upregulation of FGFR2c in our AK samples could be accompanied by a modulation of CSL. To address this point, CSL expression levels were evaluated, coupled with those of the pro-autophagic kinase ULK3, which is negatively regulated by CSL and whose increase is a critical event for the CAF program activation [23]. Molecular analysis revealed a clear repression of CSL (Figure 1B, upper panel) and an induction of ULK3 (Figure 1B, lower panel) in all the differently graded AK lesions, including KIN I. In addition, the analysis by the Wilcoxon test considering the entire cohort of AK samples revealed that the opposite trend of CSL and ULK3 genes was statistically significant (Figure 1B, right panels). Therefore, the upregulation of FGFR2c, visible already starting from early graded KIN I lesions, accompanies the unbalance of the CSL/ULK3 loop, making it reasonable to suppose its possible involvement in it.

**Figure 2:**
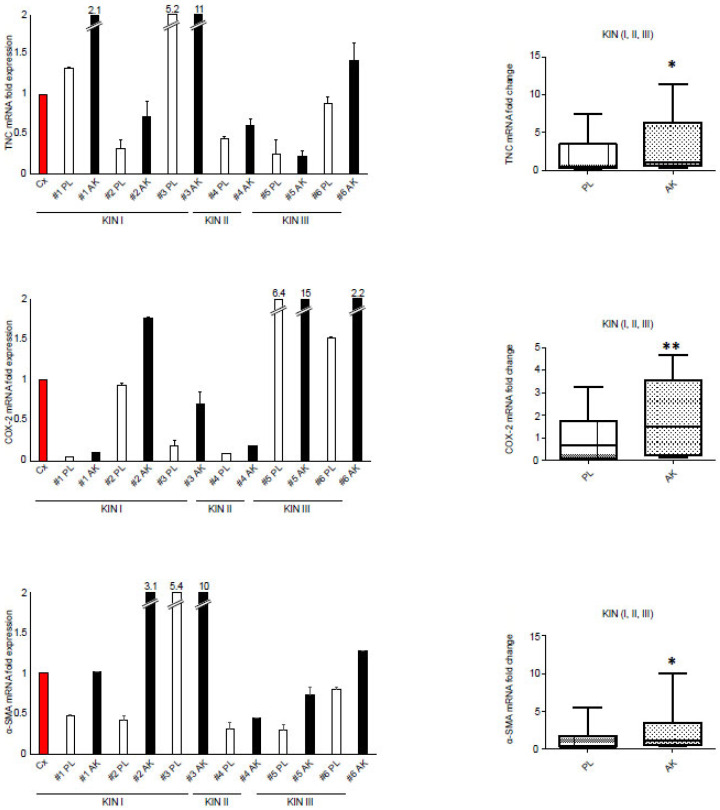
Expression of CAF genes in the dermal portion of KIN I, KIN II, and KIN III samples. TNC, COX-2, α-SMA mRNA levels were evaluated by real-time RT-PCR in KIN I, KIN II, and KIN III samples (PL—perilesional samples, white bars; AK—lesional samples, black bars) and normalized with respect to the mean of all the perilesional values (Cx, red bar), as reported in the graph. Results are expressed as mean ± standard deviation (SD). Statistical analysis of relative expression rates of KIN I + KIN II + KIN III AK versus KIN + KIN I + KIN III PL samples was performed using Wilcoxon rank-sum test, and significance levels were defined as *p* < 0.05. * *p* < 0.05; ** *p* < 0.01 versus the corresponding PL samples.

We further assessed the CAF-like signature in our sample cohort, focusing on the expression of COX-2, α-SMA, and TNC CAF genes, whose induction depends on CSL downregulation [15] and on the consequent induction of ULK3, which, in turn, activates CAF program via the transcription factor GLI 2 [23]. Molecular analysis revealed that, in all differently graded KIN lesions, the expression of all the considered CAF genes was significantly increased compared to the corresponding perilesional controls (Figure 2).

**Figure 3:**
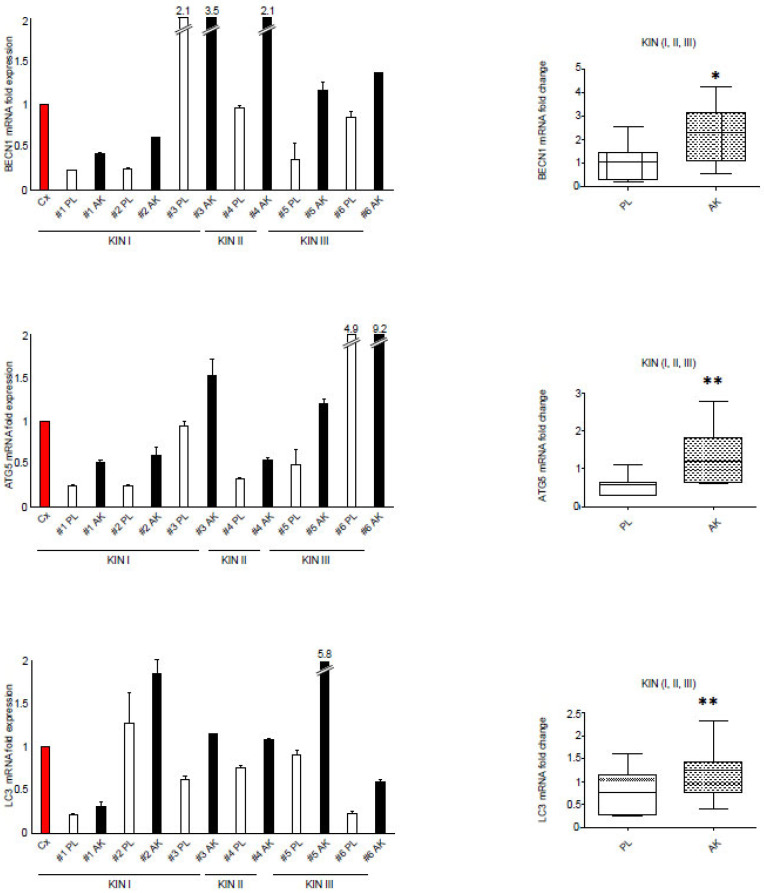
Expression of autophagic genes in the dermal portion of KIN I, KIN II, and KIN III samples. BECN1, ATG5, and LC3 mRNA levels were evaluated by real-time RT-PCR in KIN I, KIN II, and KIN III samples (PL—perilesional samples, white bars; AK—lesional samples, black bars) and normalized with respect to the mean of all the perilesional values (Cx, red bar) as reported in the graph. Results are expressed as mean ± SD. Statistical analysis of relative expression rates of KIN I + KIN II + KIN III AK versus KIN + KIN I + KIN III PL samples was performed using Wilcoxon rank-sum test, and significance levels were defined as *p* < 0.05. * *p* < 0.05; ** *p* < 0.01 versus the corresponding PL samples.

In addition to ULK3, we also analyzed the expression of other key autophagic genes, such as BECN1, ATG5 and LC3, whose expression appeared significantly upregulated in all differently graded lesions, including KIN I (Figure 3). The results reported so far are in line with previous findings, reporting the pivotal role of CSL/ULK3 negative loop in the simultaneous regulation of autophagy and CAF program activation [23,24], both events required for tumor-enhancing properties of stromal cells.

**Figure 4:**
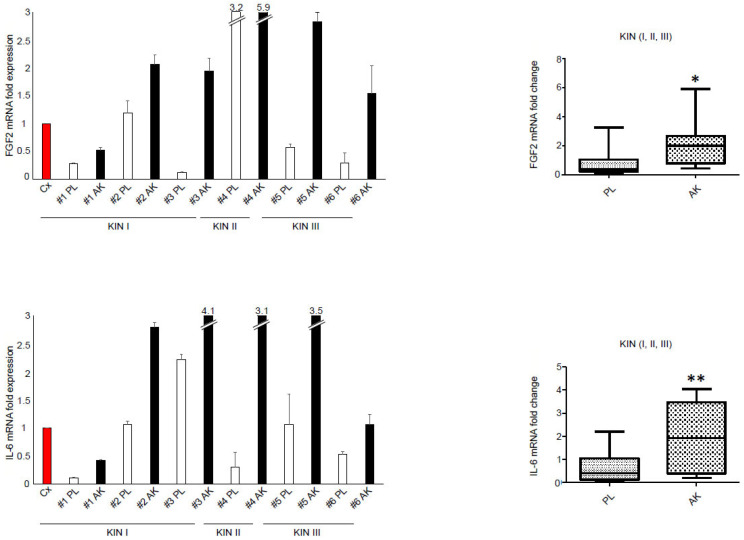
Expression of cancer-promoting factors in the dermal portion of KIN I, KIN II, and KIN III samples. FGF2, IL-6 mRNA levels were evaluated by real-time RT-PCR in KIN I, KIN II, and KIN III samples (PL—perilesional samples, white bars; AK—lesional samples, black bars) and normalized in respect to the mean of all the perilesional values (Cx, red bar), as reported in the graph. Results are expressed as mean ± SD. Statistical analysis of relative expression rates of KIN I + KIN II + KIN III AK versus KIN + KIN I + KIN III PL samples was performed using Wilcoxon rank-sum test, and significance levels were defined as *p* < 0.05: * *p* < 0.05; ** *p* < 0.01 versus the corresponding PL samples.

We then analyzed the trend of cancer-promoting factors involved in tumor/stroma crosstalk, such as FGF2 [8,15,20], and IL-6, this last systemically elevated in HNSCC patients [25], linked to resistance to therapy [26] and secreted from stromal fibroblasts in several cancer types [27]. The molecular analysis revealed a significant increase not only in FGF2, as expected [15], but also in IL-6, in all the differently graded lesions, including KIN I lesions (Figure 4).

### 3.2. The Increased Expression of FGFR2c Contributes to the Enhancement of the Autophagic Process and CAF Gene Induction in KIN I-Derived Fibroblasts

Since FGF2 upregulation triggers the FGF2/FGFR-mediated autophagic process in stromal cells, which significantly contributes to the release of tumor-promoting factors, including IL-6, during HNSCC malignant progression [20], we wondered if a comparable autophagic mechanism could also contribute to KIN I progression and if FGFR2c could upstream be involved in it. To address this topic, we first tried to determine whether the observed transcriptional induction of the autophagic genes detected in the entire dermal portion of AK lesions (see Figure 3) effectively results in an enhancement of the autophagic membrane trafficking in the fibroblast component of the stroma and whether this phenomenon could depend on FGFR2c expression rate in these specific cells. To this aim, the investigation was shifted to the in vitro model of primary HFs, selecting two cultures derived from KIN I dermal samples presenting different levels of FGFR2c expression, as well as very low (#1) and very high (#2) divergence between perilesional (PL) and lesional (AK) expression of this receptor, respectively (refer to Figure 1A). In addition, given the complexity of the dermal context, the shifting to the isolated primary cultures of HFs has been crucial to assess whether the molecular traits found in the dermal samples can be ascribed to this specific cell type, excluding other cellular compounds, such as inflammatory cells, that can differently infiltrate the sample, independently from histopathological grading.

**Figure 5:**
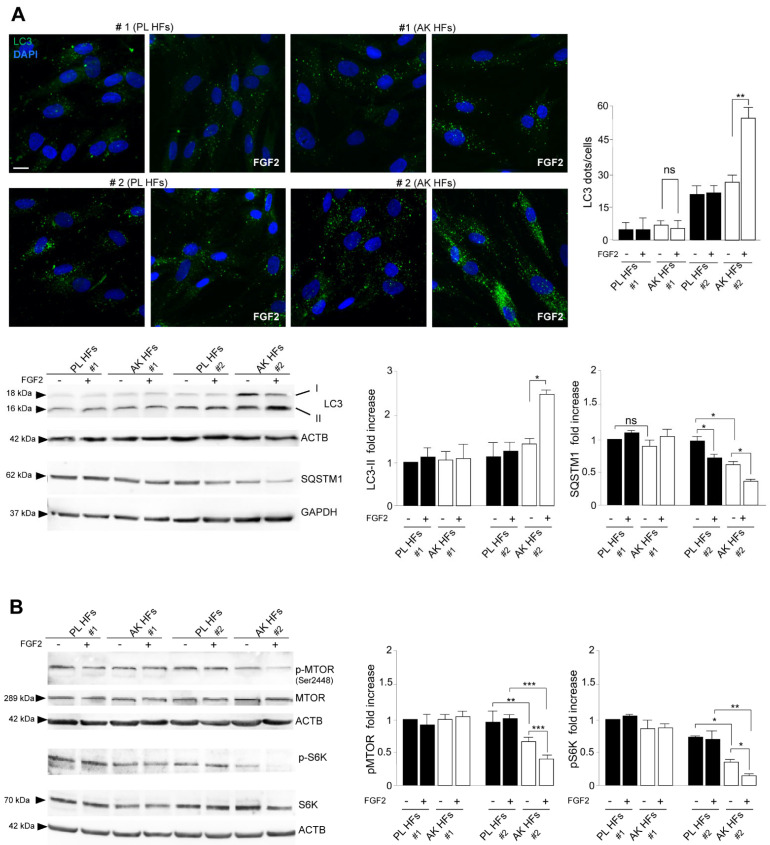
The enhancement of autophagy in response to FGF2 take place only in KIN I-derived HFs, highly expressing FGFR2c, and is driven by MTOR-signaling repression. Primary cultures of HFs isolated from perilesional (PL) or lesional (AK) samples of patients #1 and #2 were left untreated or stimulated with FGF2, as described in Material and Methods. (**A**) Quantitative immunofluorescence analysis of LC3 signal shows that both #2AK and #2PL cultures display higher basal autophagy compared to #1 cells. A significant increase of LC3 positive dots per cell in response to FGF2 stimulation is evident only in #2AK cells (lower panel), while #1AK cells (upper panel) and both #1 and #2PL controls appear unresponsive. Quantitative analysis of LC3 positive dots per cell was performed as described in Section 2, and the results are expressed as mean values ± SD. The student’s t-test was performed, and significance levels were defined as *p* < 0.05. ** *p* < 0.01; ns not significant. Bar 10 µm. Western blot analysis shows that LC3-II levels are enhanced in response to FGF2 only in #2AK samples. Reduced basal levels of SQSTM1 were observed only in #2AK cultures compared to #2PL or to #1PL and #1AK samples. FGF2 stimulation significantly reduces the accumulation of SQSTM1 in both PL and #2AK samples. No changes of SQSTM1 levels are observed in #1PL and #1AK cells. Equal loading was assessed with anti-ACTB and anti-GAPDH antibodies. (**B**) Western blot analysis shows that all cultures display comparable levels of MTOR protein, while reduced basal phosphorylation of this protein and of S6K downstream substrate, which is further decreased after FGF2 stimulation, is observed only in #2AK culture. Equal loading was assessed with anti-ACTB antibody. For all densitometric analyses (**A**,**B**), the values from three independent experiments were normalized, expressed as fold increases, and reported as mean values ± SD. Student’s t-test was performed, and significance levels are defined as *p* < 0.05. * *p* < 0.05; ** *p* < 0.01; *** *p* < 0.001; ns not significant.

Preliminary molecular and biochemical analysis by real-time RT-PCR and Western blot confirmed that the two selected isolated cultures conserved a comparable trend in terms of FGFR2c expression. In fact, while #1AK- and #1PL-derived cells expressed comparable and very low levels of FGFR2c-specific mRNA and protein (Supplementary Figure S1A,B), #2-derived cultures expressed higher levels of this receptor, with a further significant increase in AK-derived cells (Supplementary Figure S1A,B). Even if the anti-FGFR2 antibody used for blotting does not discriminate between FGFR2 isoforms, the origin of the HF cultures from dermal specimens displaying undetectable levels of the epithelial FGFR2b isoform (refers to Figure 1A) indicated that the band detected at the molecular weight of FGFR2 correspond to the mesenchymal FGFR2c variant. These results also suggest that the trend of FGFR2c expression previously observed in the entire dermal compartment by molecular approaches can be mainly ascribed to the fibroblast component. Then, to monitor autophagy, cells were left serum starved or stimulated with FGF2, a ligand which binds several FGFRs, including FGFR2c, but not its epidermal counterpart FGFR2b. The autophagic response was monitored using the widely recognized autophagic marker LC3 by quantitative immunofluorescence analysis. The results showed that both #2AK and #2PL cultures displayed higher starvation-induced autophagy (assessed as LC3 positive dots per cell) compared to #1 cells (Figure 5), even if a significant increase of process in response to FGF2 stimulation was evident only in #2AK cells (Figure 5A). In contrast, #1AK culture (Figure 5A, upper panel) and both #1 and #2 PL controls appeared quite unresponsive to FGF2 (Figure 5A). Parallel Western blot analysis confirmed the immunofluorescence observations, showing an increase of lipidated LC3 (LC3-II) levels in response to FGF2 exclusively in AK#2 sample (Figure 5A). In addition, reduced basal levels of the autophagy substrate SQSTM1 were particularly observed in unstimulated #2AK culture compared to its PL counterpart or to both #1PL and #1AK samples (Figure 5), while FGF2 stimulation significantly reduced SQSTM1 accumulation in both PL and #2AK samples, but not in #1 cultures (Figure 5A). Thus, in KIN I lesions, the responsiveness to FGF2 in terms of enhancement of autophagic membrane trafficking and flux appears to be appreciable in those stromal cells highly expressing FGFR2c.

Since previous findings demonstrated that, in advanced HNSCC, secretory autophagy is activated by FGF2/FGFR-dependent transcriptional repression of mTOR [28], we wondered if this mechanism could also take place in the context of KIN I lesions. Western blot analysis showed that, independently from FGF2 stimulation, all cultures from #1 and #2 KIN I samples displayed comparable levels of MTOR protein (Figure 5B), suggesting that this substrate is not transcriptionally modulated. However, reduced basal phosphorylation at the activating site of ser2448 of MTOR as well as of its downstream substrate S6K (at ser 371), which were further decreased after FGF2 stimulation, was observed only in #2AK culture (Figure 5B). Thus, the previously observed enhancement of autophagy induced by FGF2 in these cells appears to be possibly mediated by MTOR-signaling shut-off. Moreover, since this functional impairment of MTOR is evident only in #2AK culture and appeared enhanced by FGF2 stimulation, it is reasonable to suppose its possible dependence on the high expression and activation of FGFR2c.

**Figure 6:**
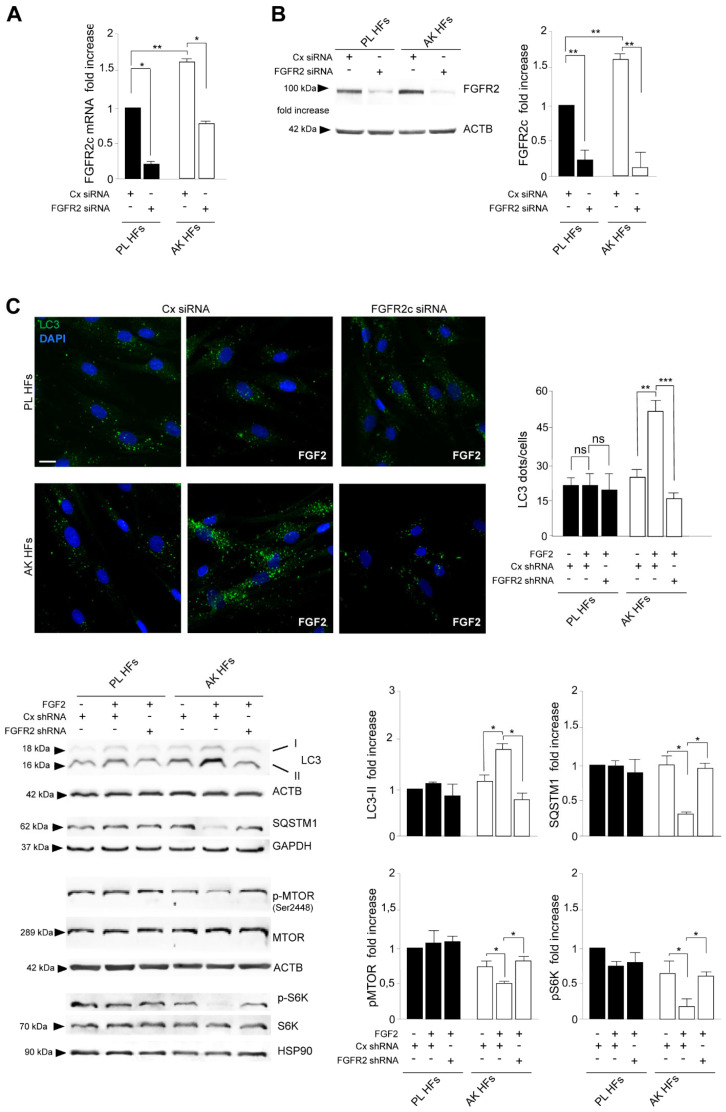
High expression of FGFR2c in KIN I-derived HFs is responsible for the increase of the autophagic process in response to FGF2. Primary culture of HFs isolated from #2PL and #2AK samples were transfected with FGFR2c siRNA and stimulated or not with FGF2, as reported in material and methods. Unrelated siRNA (Cx siRNA) was used as negative control. Real-time RT-PCR analysis (**A**) and Western blot analysis (**B**) display the efficiency of FGFR2c depletion by siRNA. Equal loading was assessed with the anti-ACTB antibody. Densitometric analysis was assessed as reported in Figure 5. Student’s t-test was performed, and significance levels are defined as *p* < 0.05. * *p* < 0.05; ** *p* < 0.01. (**C**) Quantitative immunofluorescence analysis of LC3 signal shows that the increase of LC3 dots, observed in AK cells in response to FGF2, is counteracted by FGFR2c silencing. Quantitative analysis of LC3 positive dots per cell was performed as described in Section 2, and the results are expressed as mean values ± SD. Student’s t-test was performed, and significance levels have been defined as *p* < 0.05. ** *p* < 0.01; *** *p* < 0.001; ns not significant, bar 10 µm. Western blot analysis shows that the increase of LC3-II levels and the decrease of SQSTM1 in #2AK cells in response to FGF2, as well as the reduction of MTOR and S6K phosphorylation in both unstimulated and FGF2-stimulated #2AK cells, are counteracted by FGFR2c silencing. Equal loadings were assessed with anti-ACTB, anti-GAPDH, and anti-HSP90 antibodies. Densitometric analysis was assessed as reported above. Student’s t-test was performed, and significance levels are defined as *p* < 0.05. * *p* < 0.05.

**Figure 7:**
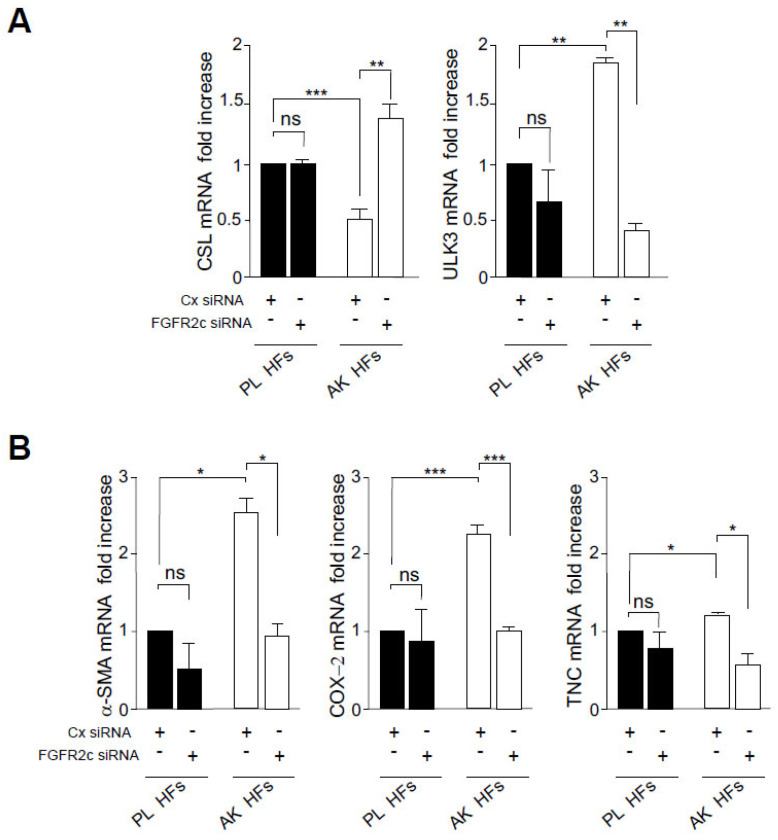
Highly expressed FGFR2c triggers CAF gene induction in AK KIN I-derived HFs. Primary cultures of HFs isolated from #2PL and #2AK samples were transfected with FGFR2c siRNA and Cx siRNA as above. Real-time RT-PCR analysis reveals a repressive effect of FGFR2c depletion on the increase of ULK3 and on the repression of CSL (**A**), as well as on the more pronounced expression of CAF genes (**B**) displayed by AK cultures compared to their corresponding controls. Results are expressed as mean values ± SD. The student’s t-test was performed, and significance levels are defined as *p* < 0.05. * *p* < 0.05; ** *p* < 0.01; *** *p* < 0.001; ns not significant.

To demonstrate that the enhancement of the autophagic process in response to FGF2, detected only in #2AK culture, can be ascribed to the observed high expression of FGFR2c, we analyzed the effects of receptor depletion by small interfering RNA approaches, whose efficiency was in advance assessed by real-time RT-PCR and by Western blot analysis (Figure 6A,B). The autophagic process was analyzed by immunofluorescence and by biochemical approaches as above, which demonstrated that the increase of LC3 dots per cell and of LC3-II levels, as well as the decrease of SQSTM1 substrate, observed in #2AK cells in response to FGF2 (Figure 6C), was significantly counteracted by FGFR2c silencing (Figure 6C). Moreover, the evident reduction of MTOR and S6K phosphorylation observed in both unstimulated and FGF2-stimulated #2AK cells compared to the corresponding PL controls, appeared impaired by the receptor depletion (Figure 6C), strengthening the hypothesis of its dependence on FGFR2c expression levels and activation. In addition, the molecular analysis revealed a counteracting effect of FGFR2c depletion also on the repression of CSL (Figure 7A) and on the induction of ULK3 (Figure 7A), as well as on the more pronounced CAF signature (Figure 7B) observed in #2AK cultures compared to their corresponding controls. It is interesting to note that the use of the isolated primary cultures once again allowed us to determine that the expression trend of the mentioned genes previously assessed in the dermal samples can be ascribed to the stromal component of fibroblasts. Overall, the results suggest that, when highly expressed, FGFR2c appears to contribute to precocious CAF gene induction, as well as to the enhancement of the autophagic trafficking: two linked tumor-promoting events controlled the CSL/ULK3 loop and were crucial in determining the tumor/stromal crosstalk.

### 3.3. The Enhancement of IL-6 Secretion in KIN I-Derived Fibroblasts Can by Ascribed to FGFR2c High Expression and Signaling

**Figure 8:**
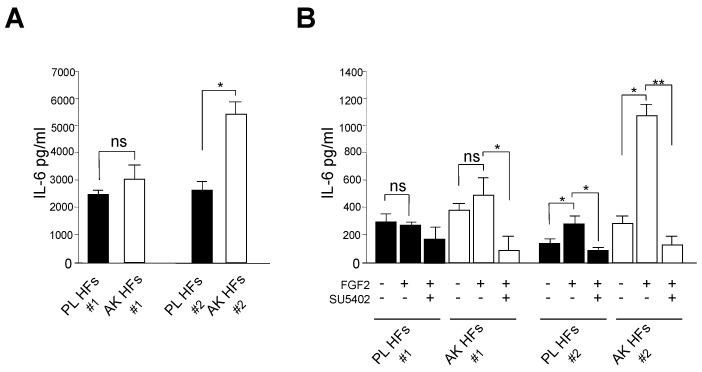
In AK KIN I-derived HFs, the increased secretion of IL-6 depends on FGFR2c high expression and signaling. Primary cultures of HFs from #1PL, #1AK, #2PL, and #2AK samples were left in serum (**A**) or serum starved and stimulated with FGF2 in the presence or not of the SU5402 (**B**). Magnetic bead-based multiplex assay performed on cell supernatants (SN) shows that the IL-6 protein levels from serum-fed #2AK cells are significantly higher compared to those detected in the SN from the corresponding PL controls or from both #1AK and #1PL cultures (**A**). In #2AK cells, the levels of IL-6 released in the SN are significantly increased by FGF2 stimulation but strongly dampened by SU5402 (**B**). The values are expressed in pg/mL. Results are expressed as mean values ± SD. The student’s t-test was performed, and significance levels are defined as *p* < 0.05. * *p* < 0.05; ** *p* < 0.01; ns not significant.

Encouraged by the data obtained in HNSCC, indicating a central role of FGFR-mediated autophagy in enhancing the release of tumor-promoting factors by stromal cells [20], we finally aimed to assess if the increase of autophagy observed in #2AK cultures could result in an increased secretion of IL-6. A magnetic bead-based multiplex test demonstrated that the protein levels in the supernatant (SN) from serum-fed #2AK cells were significantly higher compared to those detected in the SN from the corresponding PL controls or from both, #1AK and #1PL cultures (Figure 8A). To directly link IL-6 secretion to FGFR2c signaling, cells were left serum starved or stimulated with FGF2 as above in the presence or not of the FGFR2 kinase inhibitor SU5402. Interestingly, the magnetic bead-based assay showed that, especially in the #2AK culture, IL-6 levels in SN were significantly increased by FGF2 stimulation (Figure 8B) but strongly dampened by SU5402 (Figure 8B). Even if it has been reported that SU5402 can target other FGFRs and RTKs (such as PDGFR and VEGFR), the coupling of the induction by FGF2 and the inhibition by SU5402 suggested that the improvement of IL-6 secretion can be attributed to the activity of FGFR family members. Furthermore, the evidence of an almost comparable level of FGFR1, FGFR3, and FGFR4 expression in the used cells (Supplementary Figure S2) strengthened the possibility of dependence on FGFR2c expression levels and signaling.

Overall, our data appear to suggest the possibility that the upregulation of FGFR2c in KIN I fibroblasts could contribute not only to a CSL/ULK3-dependent precocious CAF and autophagic signature but also to an effective enhancement of autophagic membrane trafficking, which possibly might underly the increased release of tumor-promoting factors, such as IL-6.

## 4. Discussion

Actinic keratosis (AK) is a preneoplastic skin disease associated with dysregulated UV exposure, which sometimes can rapidly progress to aggressive SCCs, through an alternative pathway, named the “differentiated” way. This kind of pathway involves the direct progression of early KIN I lesions to cutaneous squamous cell carcinomas (SCC) [3,5] and presents typical hallmarks, such as EMT [6].

Cutaneous SCC is the most common non-melanoma skin cancer (NMSC) [29], whose incidence is on the rise [30] due to the increase of excessive and unprotected sun exposure, coupled with an increase in immunosuppressive approaches [31]. In light of this evidence, identifying the early molecular drivers and the dysregulated signaling pathways acting in precancerous AK and SCCs is becoming a pivotal goal for the development of therapeutic and prevention strategies.

Accumulating findings pointed to the dysregulation of the fibroblast growth factor receptor (FGFR) axis as an additional event contributing to AK pathogenesis [7,8,9]. In line with this and with our previous evidence pointing to FGFR2 isoform switch as a key upstream event in early epidermal carcinogenesis and EMT induction [12,13,14,15], we recently proposed that FGFR2 altered splicing and the consequent aberrant expression of the mesenchymal FGFR2c variant, changing keratinocyte response to paracrine FGFs, could contribute to AK rapid malignant progression [15]. The additional observation of an increased expression of FGF2 in the dermal portion of some AK lesions also suggested the establishment of aberrant tumor/stromal loops based on the FGF2/FGFR2c axis [15].

Indeed, it is now widely accepted that tumor stromal cell dysregulations, particularly those involving cancer-associated fibroblasts (CAF) [32], strongly contribute to carcinogenesis [33,34] prevalently via the establishment of the altered, pro-tumorigenic tumor/stromal loops. In addition, accumulating observations have suggested that CAFs are a functionally heterogeneous population [35] deriving from multiple cell types, including normal stromal fibroblasts and tumor cells that underwent EMT [32,36]. However, even if the phenotypical hallmarks distinctive of the different CAFs subgroups in several tumors become characterized [35], the molecular drivers and signaling pathways involved in normal fibroblast conversion into CAFs, as well as for CAF expansion and diversification, and in particular the complexity of their multiple crosstalks, still remain unclear.

In this work, we found that KIN I-derived dermal portions display a variable FGFR2c upregulation, accompanied by a CAF signature (indicated by CSL repression, ULK3 upregulation and the induction of the CAF genes TNC, α-SMA and COX-2) and by a significant increase of autophagic genes and tumor-promoting factors, such as FGF2 and IL-6. This observation suggested the possibility that the CSL/ULK3 negative loop in stromal cells and its downstream-dependent processes (CAF gene activation and transcriptional induction of autophagy) could be early events in AK progression, possibly upstream controlled by FGFR2c and in close dependence on its expression. Our idea was in line with previous studies pointing to the FGF2/FGFR axis as an essential element for CAF activation in advanced HNSCC [20] and for CSL-dependent CAF expansion in AK and cutaneous SCC [8]. The novelty of our hypothesis was the identification of the mesenchymal FGFR2c variant as a possible preeminent FGFR family member involved. In apparent contrast with us, recent works have indicated the TGF-β [18] and other TGF-β family members [19] as major players in CAF differentiation. Indeed, Bordignon and co-workers found that the FGF2/FGFR1 and TGF-β/TGFBRII axes exert alternative effects, resulting in fibroblast differentiation in EMT-promoting and inflammation-promoting CAFs, respectively. However, these authors worked in normal fibroblasts while our data refer to pre-neoplastic AK fibroblasts, pointing to FGF2/FGFR2c axis as a key event which we suppose can possibly act in synergy with TGF-β/TGFBRII signaling in inducing EMT-promoting CAF phenotype. In any case, these observations strengthen the idea that, during carcinogenesis, the expression pattern of the different FGFR family members can progressively change over time, modifying cellular responses to microenvironmental factors. This is true not only for neoplastic cells but also for cancer-associated stromal cells, contributing to the establishment of tumor plasticity.

In the second step of our work, taking advantage of the use of primary cultures of HFs derived from two specifically selected KIN I samples, we demonstrated that only KIN I cultures expressing high levels of FGFR2 responded to FGF2 in terms of increased autophagic membrane trafficking and flux. Gene-silencing approaches by siRNA indicated that the observed enhancement of autophagy, as well as the increase of CAF signature, can depend on the FGFR2c upregulation level. Additionally, in light of the context of advanced HNSCC [28], we also found that the functional shut-off of the MTOR pathway downstream of FGFR2c is the main pathway possibly involved. Investigating either the activation of FGFR2c early signaling or the time kinetics of mTOR phosphorylation after FGF2 short-term stimulation are additional important points to further clarify FGFR2c function in AK-derived HFs. However, to better mimic the “in vivo” availability of paracrine factors, we limited all our “in vitro” experiments to single, prolonged stimulation with FGF2; therefore, suitable experiments for FGFR2c early activation will be performed in the next future, possibly extending our patient cohort. It is worth also remarking that the use of the isolated cultures of HFs also allowed us to assess the upregulation of FGFR2c, as well as the modulation of the CSL/ULK3 loop and of CAF genes, previously observed by us analyzing the entire dermal portion of AK lesions, is specifically attributable to these stromal cells. Finally, a magnetic bead-based multiplex assay, performed on SNs from KIN I-derived HF primary cultures treated or not with the FGFR2 kinase inhibitor SU5402, indicated that the secretion of the tumor-promoting factor IL-6 is enhanced when FGFR2c is upregulated and activated, and we speculated that an FGFR2c-dependent increase of autophagy could contribute to this phenomenon. To strengthen our hypothesis, in our next work, the effects of autophagy inhibition on IL-6 secretion will be investigated, using HF primary cultures derived from a larger cohort of AK patients.

**Figure 9:**
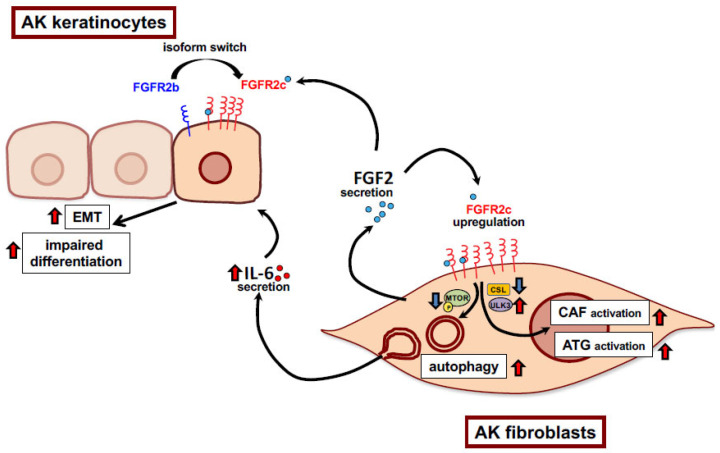
Schematic drawing of FGFR2c axis in the regulation of precancerous/stroma AK cell crosstalk.

Although the restricted number of our sample cohort makes us cautious in drawing conclusions, the data obtained on the in vitro model of primary cultures of dermal fibroblasts suggest the idea that both secretory autophagy (which can implement tumor-promoting factor release) and CAF program activation in stromal fibroblasts not only contribute to SCC malignant progression, as previously described [20], but can also take place in precancerous AK lesions, where they could be upstream regulated by overexpressed FGFR2c. In light of our previous findings indicating a possible oncogenic role for FGFR2c, when aberrantly expressed in the epithelial counterpart of AK lesions [15], we can conclude that the FGFR2c axis could represent a tumor-promoting event in both precancerous AK keratinocytes and stromal cells, contributing to their oncogenic crosstalk (Figure 9). Further investigations will be needed to definitively establish whether this receptor could actually represent an important molecular marker for the identification of those early AK lesions destined to a rapid malignant progression.

## Data Availability

All the data is provided in the article.

## Supplementary Materials

The following supporting information can be downloaded at: https://www.mdpi.com/article/10.3390/biology12030463/s1, Figure S1: FGFR2c expression in #1- and #2-derived primary cultures of HFs; Figure S2: FGFR1c, FGFR2c, and FGFR4 expression in #1- and #2-derived primary cultures of HFs; Figures S3–S6: Full Western blot images for Figure 5A; Figures S7–S12: Full Western blot images for Figure 5B; Figures S13 and S14: Full Western blot images for Figure 6B; Figures S15–S24: Full Western blot images for Figure 6C; Figures S25 and S26: Full Western blot images for Figure S1.
