# Supplementary material for: FGFR2c Upregulation Contributes to Cancer-Associated Fibroblast Program Activation and to Enhanced Autophagy in Actinic Keratosis-Derived Dermal Fibroblasts: A Possible Role in Precancerous Cell/Stromal Cell Crosstalk
Source: Biology (Basel). 2023 Mar 16;12(3):463. doi: 10.3390/biology12030463 (PMC10045898; doi:10.3390/biology12030463)
Supplement: Supplementary file 1 [file biology-12-00463-s001.zip › Figure S1 REVISED.pdf]

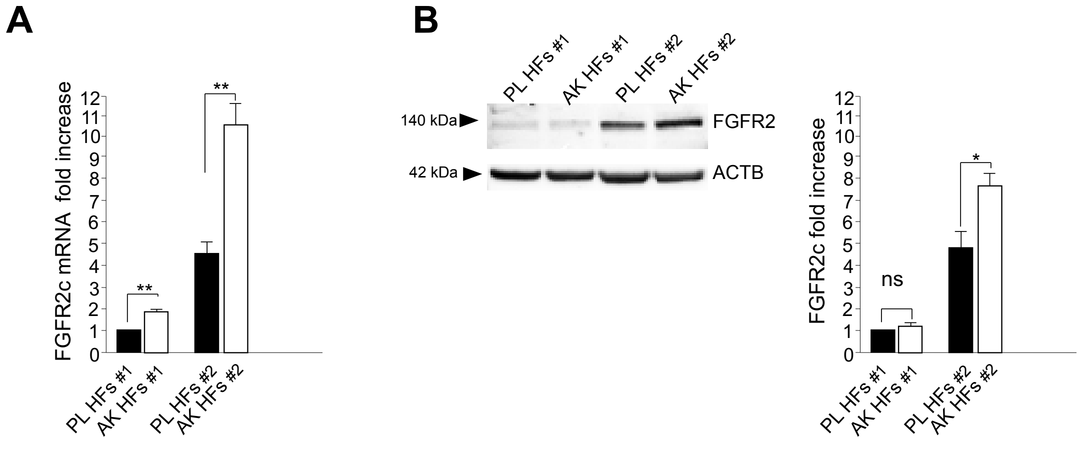

Supplementary Figure S1

FGFR2c expression in #1-derived and #2-derived primary cultures of HFs. Primary cultures of HFs from dermal portion of KIN I #1 and KIN I #2 samples were selected for molecular and biochemical analysis of FGFR2 expression. A) Real time RT-PCR performed using FGFR2c specific RNA p rimers shows that #1AK and #1PL -derived cells express comparable and very low levels of FGFR2c specific m RNA, w hile # 2-derived c ultures e xpressed h igher levels of this FGFR2 receptor variant mRNA, with and a significant i ncrease i n AK-derived cells. Results are expressed as mean values ± SD. The student's t test was performed, and significance l evels a re d efined as p< 0.05. ** p<0.01. B) Western blot analysis shows a weak band at the molecular weight corresponding to FGFR2 visible in both PL and AK #1 cells, while a more consistent band is observed in #2 cultures, whit a further increase in AK #2 cells. Equal loading was assessed with anti-ACTB. For densitometric analysis the values from three independent experiments were normalized, expressed as fold increases, and reported as mean values ± SD. Student's t test was performed, and significance l evels a re d efined as p< 0.05. * p< 0.05; ns not significant.
