# Supplementary material for: FGFR2c Upregulation Contributes to Cancer-Associated Fibroblast Program Activation and to Enhanced Autophagy in Actinic Keratosis-Derived Dermal Fibroblasts: A Possible Role in Precancerous Cell/Stromal Cell Crosstalk
Source: Biology (Basel). 2023 Mar 16;12(3):463. doi: 10.3390/biology12030463 (PMC10045898; doi:10.3390/biology12030463)
Supplement: Supplementary file 1 [file biology-12-00463-s001.zip › Figure S2 New.pdf]

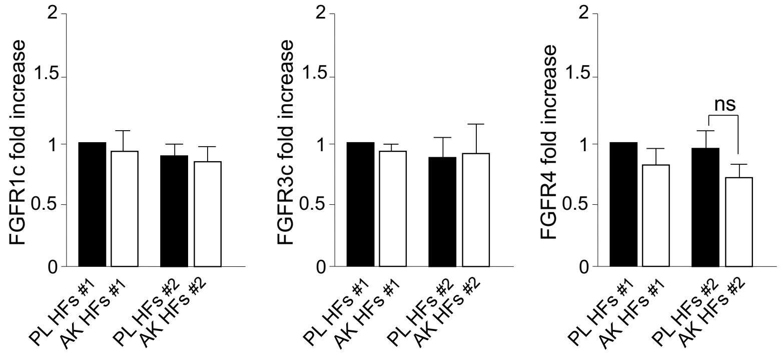

Supplementary Figure S2

FGFR1c, FGFR3c and FGFR4 expression in #1-derived and #2-derived primary cultures of HFs.

Real time RT-PCR, performed in primary cultures of HFs from dermal portion of KIN I #1 and KIN I #2 samples using specific RNA primers, shows that all analyzed cells express comparable levels of FGFR1c, FGFR3c and FGFR4 mRNAs, Results are expressed as mean values ± SD. The student's t test was performed, and significance levels are defined as $p < 0.05$. ns not significant.
