# Supplementary figures and images for: FGFR2c Upregulation Contributes to Cancer-Associated Fibroblast Program Activation and to Enhanced Autophagy in Actinic Keratosis-Derived Dermal Fibroblasts: A Possible Role in Precancerous Cell/Stromal Cell Crosstalk
Source: Biology (Basel). 2023 Mar 16;12(3):463. doi: 10.3390/biology12030463 (PMC10045898; doi:10.3390/biology12030463)

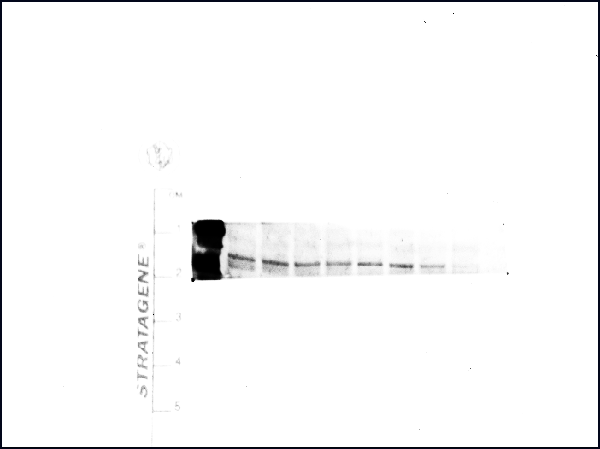

Supplement: Supplementary file 1 [file biology-12-00463-s001.zip › Figure S10 Full western Figure 5B p-S6K.tif]

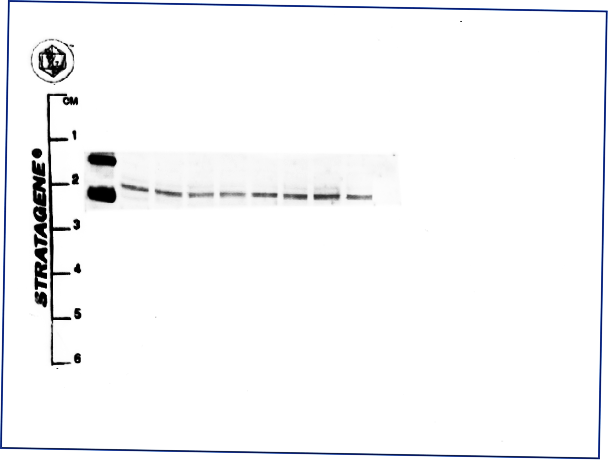

Supplement: Supplementary file 1 [file biology-12-00463-s001.zip › Figure S11 Full western Figure 5B totS6K.tif]

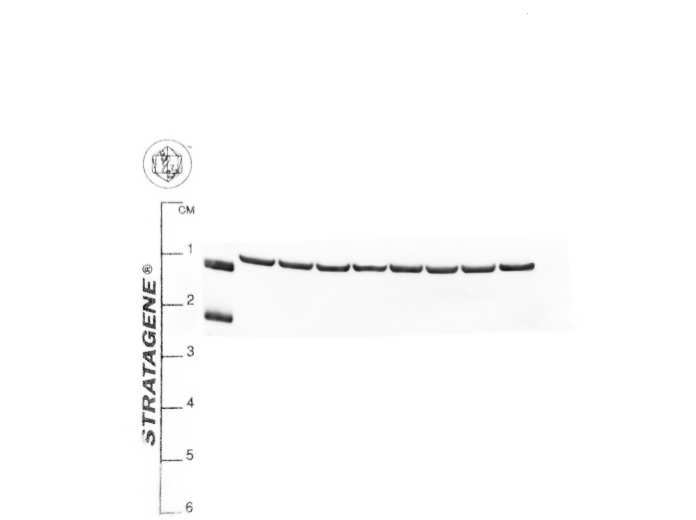

Supplement: Supplementary file 1 [file biology-12-00463-s001.zip › Figure S12 Full western Figure 5B ACTB (S6K).tif]

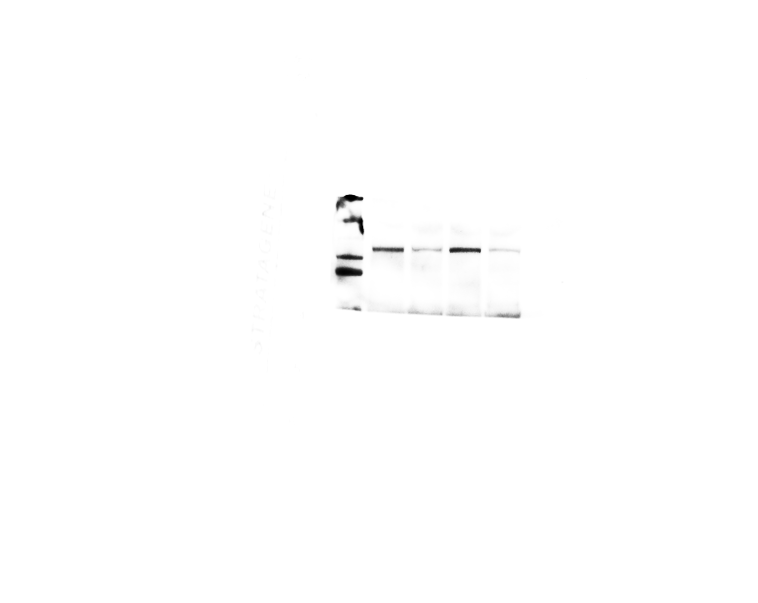

Supplement: Supplementary file 1 [file biology-12-00463-s001.zip › Figure S13 Full western Fig 6B FGFR2.tif]

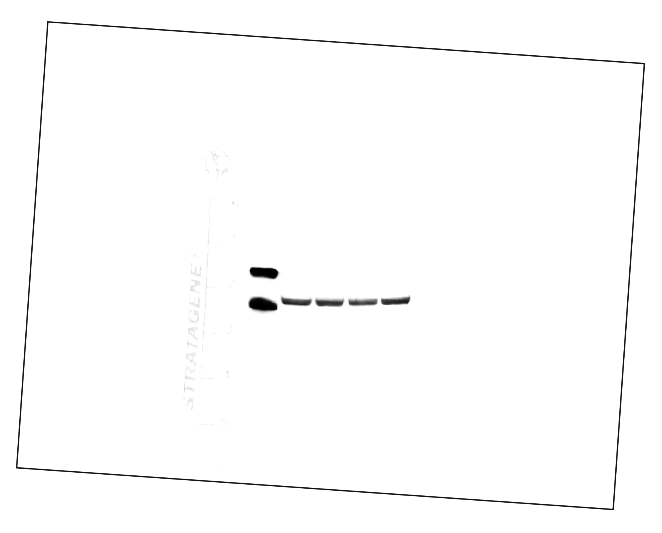

Supplement: Supplementary file 1 [file biology-12-00463-s001.zip › Figure S14 Full western Fig 6B ACTB.tif]

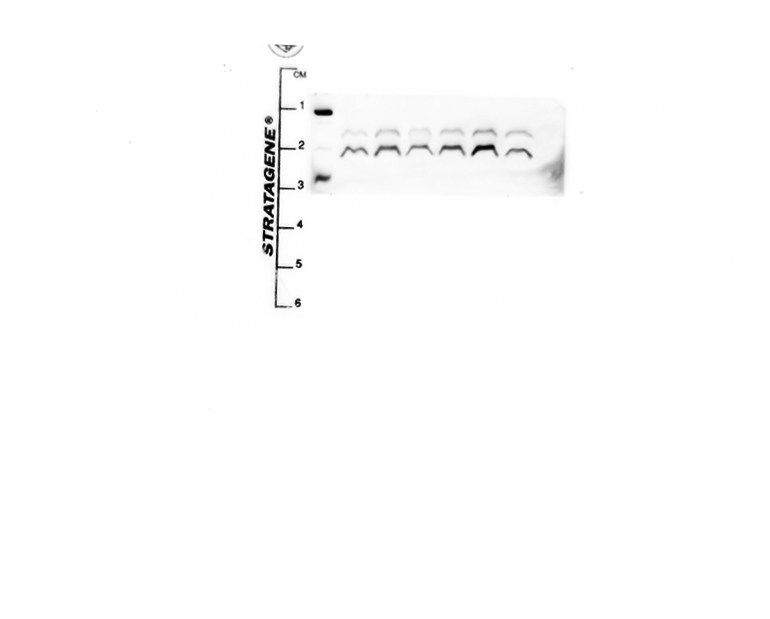

Supplement: Supplementary file 1 [file biology-12-00463-s001.zip › Figure S15 Full western Fig. 6C LC3.tif]

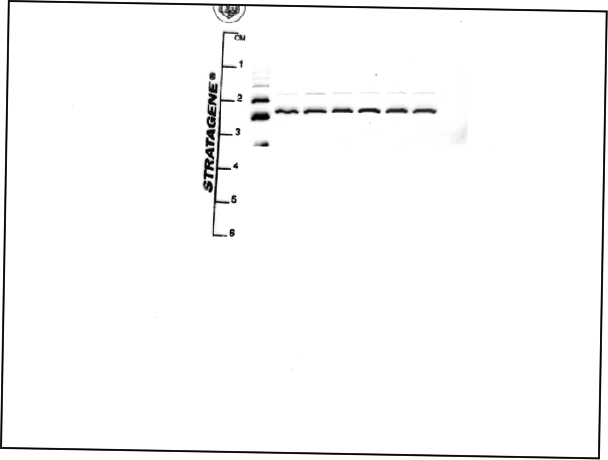

Supplement: Supplementary file 1 [file biology-12-00463-s001.zip › Figure S16 Full western Fig. 6C ACTB.tif]

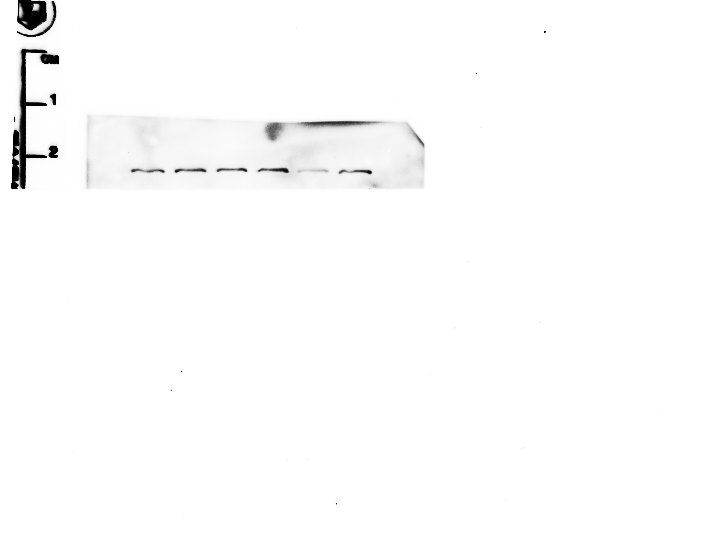

Supplement: Supplementary file 1 [file biology-12-00463-s001.zip › Figure S17 Full western Figure 6C SQSTM1.tif]

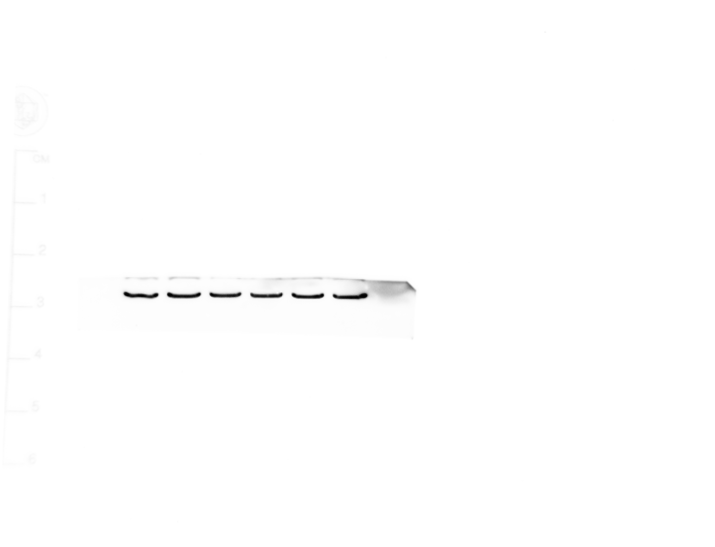

Supplement: Supplementary file 1 [file biology-12-00463-s001.zip › Figure S18 Full western Figure 6C GAPDH (SQSTM1).tif]

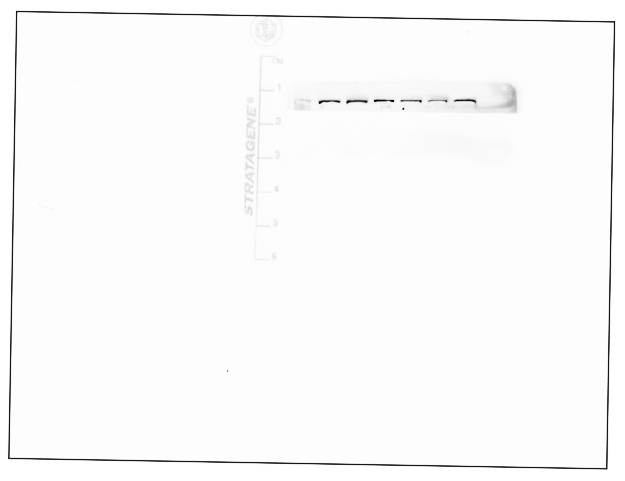

Supplement: Supplementary file 1 [file biology-12-00463-s001.zip › Figure S19 Full western Fig. 6C Phospho-MTOR.tif]

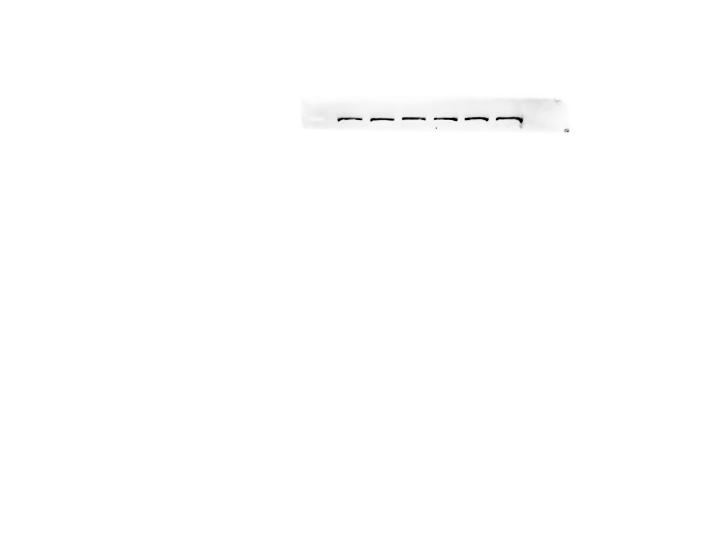

Supplement: Supplementary file 1 [file biology-12-00463-s001.zip › Figure S20 Full western Fig. 6C MTOR tot.tif]

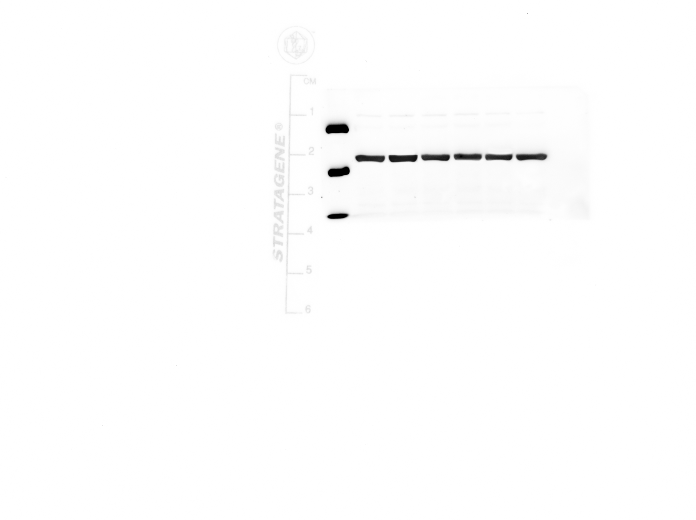

Supplement: Supplementary file 1 [file biology-12-00463-s001.zip › Figure S21 Full western Fig. 6C ACTB -MTOR.tif]

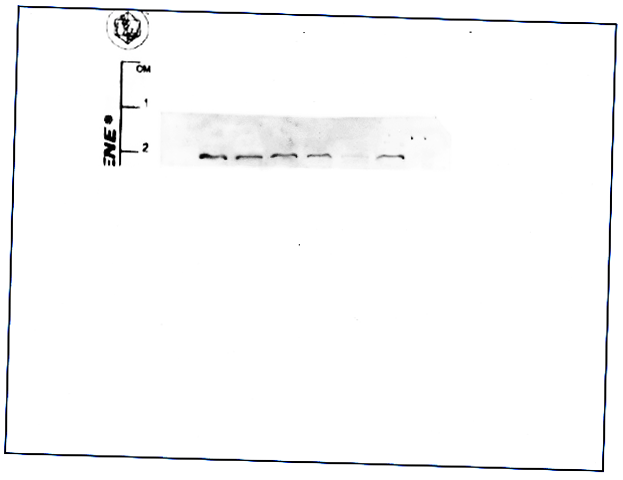

Supplement: Supplementary file 1 [file biology-12-00463-s001.zip › Figure S22 Full western Figure 6C p-S6K.tif]

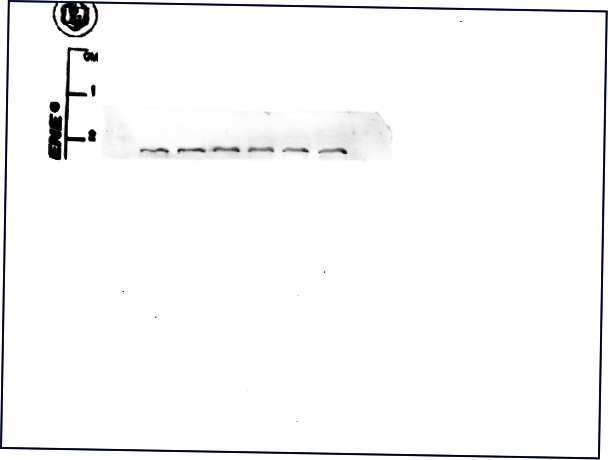

Supplement: Supplementary file 1 [file biology-12-00463-s001.zip › Figure S23 Full western Figure 6C S6K.tif]

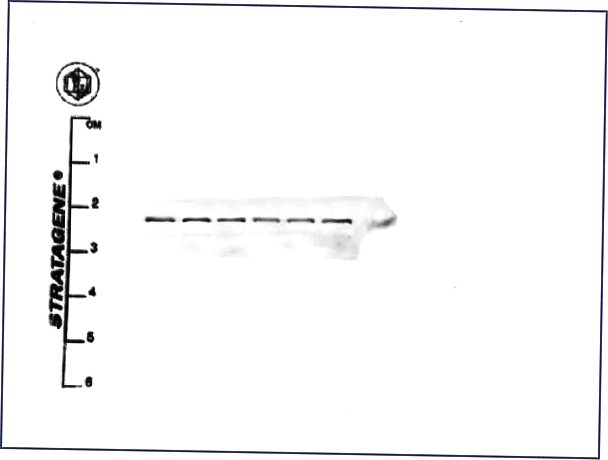

Supplement: Supplementary file 1 [file biology-12-00463-s001.zip › Figure S24 Full western Figure 6C HSP90 (S6K).tif]

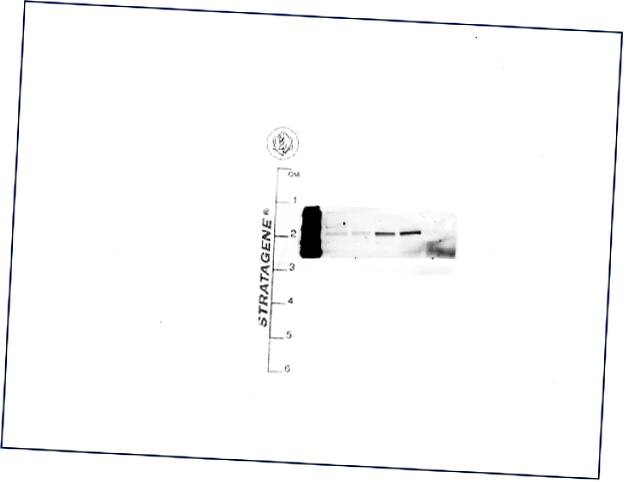

Supplement: Supplementary file 1 [file biology-12-00463-s001.zip › Figure S25 Full western SUPPLEMENTARY Fig.1 FGFR2.tif]

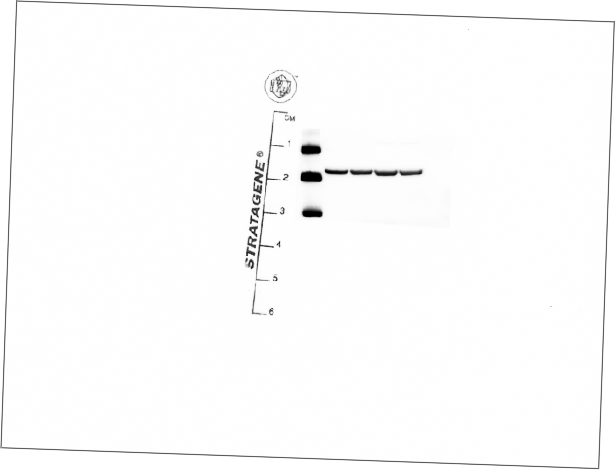

Supplement: Supplementary file 1 [file biology-12-00463-s001.zip › Figure S26 Full western SUPPLEMENTARY Fig.1 ACTB.tif]

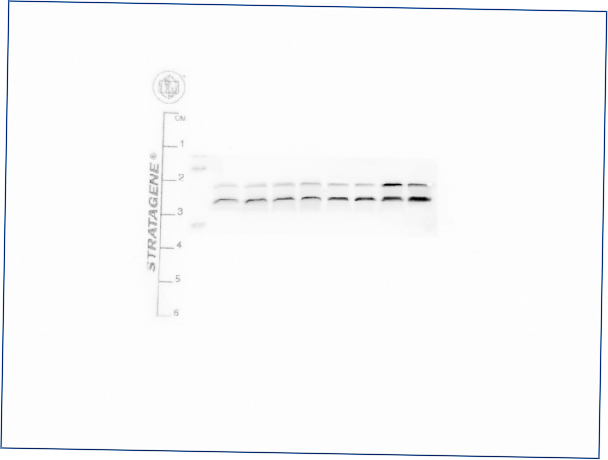

Supplement: Supplementary file 1 [file biology-12-00463-s001.zip › Figure S3 Full western Fig5A LC3.tif]

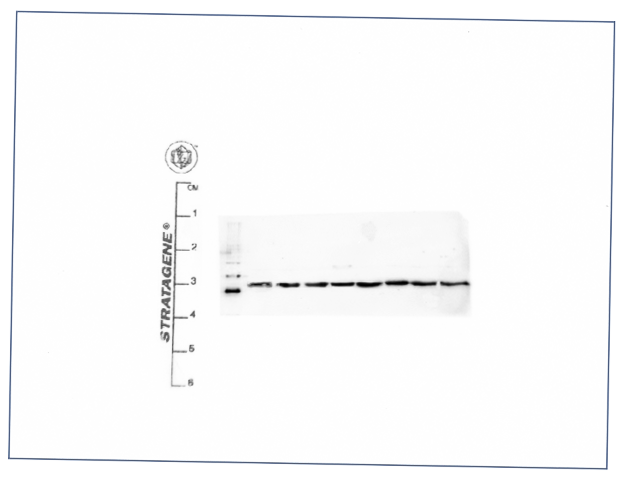

Supplement: Supplementary file 1 [file biology-12-00463-s001.zip › Figure S4 Full western Fig. 5A ATCB.tif]

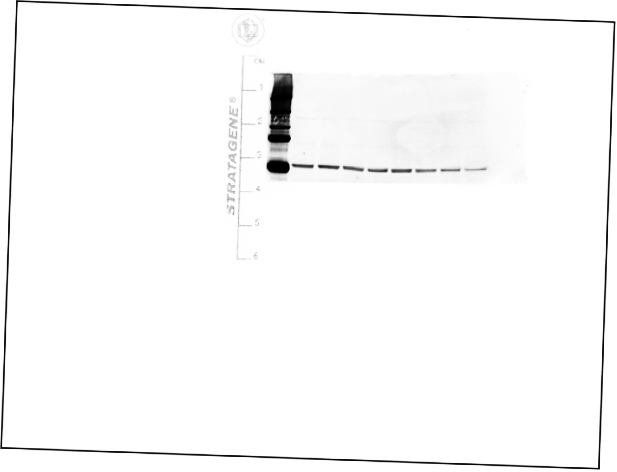

Supplement: Supplementary file 1 [file biology-12-00463-s001.zip › Figure S5 Full western Fig. 5A SQSTM1.tif]

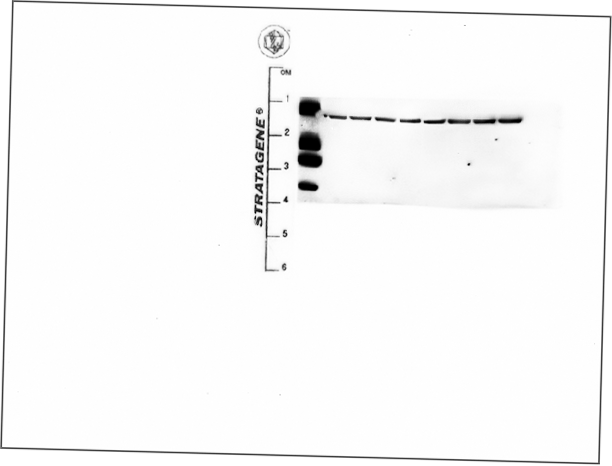

Supplement: Supplementary file 1 [file biology-12-00463-s001.zip › Figure S6 Full western Fig. 5A GAPDH.tif]

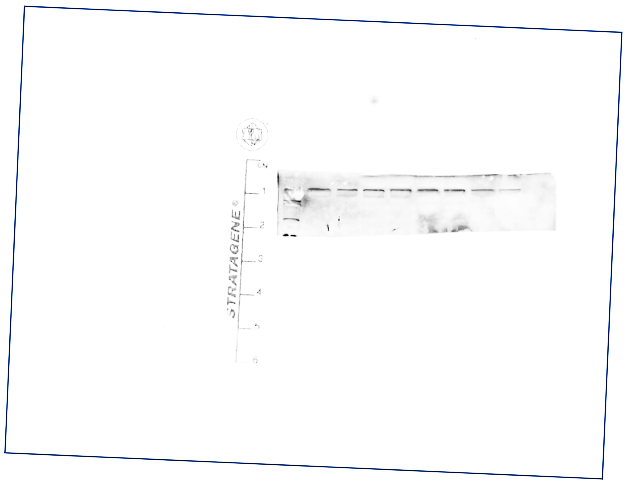

Supplement: Supplementary file 1 [file biology-12-00463-s001.zip › Figure S7 Full western Fig 5B phospho-MTOR.tif]

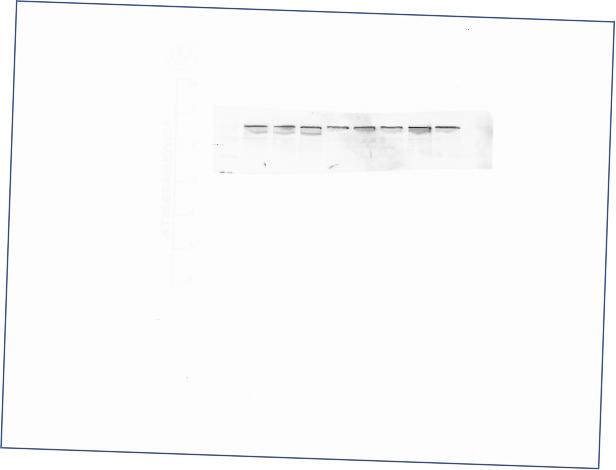

Supplement: Supplementary file 1 [file biology-12-00463-s001.zip › Figure S8 Full western Fig 5B MTOR total.tif]

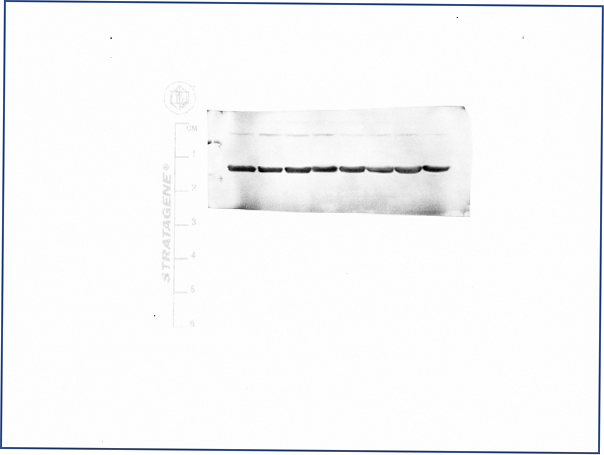

Supplement: Supplementary file 1 [file biology-12-00463-s001.zip › Figure S9 Full western Fig 5B ACTB.tif]
